# Ribonucleic acid interference knockdown of IL-6 enhances the efficacy of cisplatin in laryngeal cancer stem cells by down-regulating the IL-6/STAT3/HIF1 pathway

**DOI:** 10.1186/s12935-017-0448-0

**Published:** 2017-09-05

**Authors:** Qiang Fu, Pengruofeng Liu, Xiumei Sun, Shanshan Huang, Fengchan Han, Lili Zhang, Yannan Xu, Tingyan Liu

**Affiliations:** 1 College of Basic Medicine, Binzhou Medical University, Yantai, 264003 China; 2 Department of Stomatology, The First Affiliated Hospital, School of Medicine, Zhejiang University, Hangzhou, 310003 China; 3 Department of Otolaryngology, Yantai Affiliated Hospital of Binzhou Medical University, Yantai, 264003 China

**Keywords:** Laryngeal cancer, Cancer stem cells, Interleukin-6, siRNA

## Abstract

**Background:**

Cisplatin has been used in the treatment of many cancers, including laryngeal cancer; however, its efficacy can be reduced due to the development of drug resistance. This study aimed to investigate whether interleukin-6 (IL-6) knockdown may enhance the efficacy of cisplatin in laryngeal cancer stem cells (CSC) and the potential involvement of the signal transducer and activator of transcription 3 (STAT3) and hypoxia-inducible factor 1 (HIF1) in this effect.

**Methods:**

The ALDH+ and CD44+ CSC in Hep2 human laryngeal squamous cancer cells were identified by the fluorescence-activated cell sorting technique. IL-6, STAT3 and HIF1 mRNA and protein expressions were examined with quantitative real-time polymerase chain reaction and Western blot, respectively. Cell proliferation was measured by MTT assay. Tumorigenicity was measured by a colony formation assay and invasion was determined by a cell invasion assay. Apoptotic cells were counted by flow cytometry. Immunohistochemistry was performed to detect immunoreactive IL-6, STAT3 and HIF1 cells in xenografts.

**Results:**

The mRNA and protein levels of IL-6, STAT3 and HIF1 were significantly increased in Hep2-CSC as compared with those from Hep2 cells. Application of siRNA-IL-6 to knockdown IL-6 resulted in significantly decreased IL-6, STAT3 and HIF1 mRNA and protein levels. IL-6 knockdown reduced cell proliferation, tumorigenicity and invasion and increased apoptosis within CSC. Enhanced degrees of suppression in these parameters were observed when IL-6 knockdown was combined with cisplatin in these CSC. Results from the xenograft study showed that the combination of IL-6 knockdown and cisplatin further inhibited the growth of xenografts as compared with that obtained in the cisplatin-injected group alone. Immunoreactive IL-6, STAT3 and HIF1 cell numbers were markedly reduced in IL-6 knockdown tumor tissues. IL-6, STAT3 and HIF1 immunoreactive cell counts were further reduced in tissue where IL-6 knockdown was combined with cisplatin treatment as compared with tissue receiving cisplatin alone.

**Conclusions:**

IL-6 knockdown can increase chemo-drug efficacy of cisplatin, inhibit tumor growth and reduce the potential for tumor recurrence and metastasis in laryngeal cancer. The IL-6/STAT3/HIF1 pathway may represent an important target for investigating therapeutic strategies for the treatment of laryngeal cancer.

## Introduction

Head and neck cancers represent the seventh most common cancer worldwide [1]. In particular, head and neck squamous cell carcinoma (HNSCC) is the eighth leading cause of cancer mortality [2], with laryngeal squamous carcinoma (LSCC) being the most common type of HNSCC or head and neck cancer [3]. Chemoradiotherapy and surgery remain the major treatment modalities for head and neck cancers. Despite improvements in overall life quality achieved with the use of combined therapies, survival rates of the cancer patients have not advanced significantly over the past several decades [4]. The recurrence and metastasis of head and neck cancer are often accompanied with chemo-drug resistance generated during the cancer therapy, with the result that therapeutic outcomes are unsatisfactory.

Cancer stem cells (CSC) have become a theoretical foundation for chemo-resistance and cancer recurrence studies. CSC represent a small population of tumor cells that can uniquely self-renew, regenerate, sustain tumor growth, and thus play an important role in the growth and spread of the tumor [5–11]. During chemotherapy, CSC can mutate or experience abnormal differentiation, which may lead to tumor recurrence and metastasis and serve as the basis for drug resistance [12, 13]. Findings from recent studies have revealed that CSC can be identified and isolated through distinct cell surface markers, such as CD44 and CD133 [14–16], which are found in laryngeal carcinoma cells [8, 17]. In addition, certain intracellular protein molecules have also been used for isolating and detecting CSC. For example, aldehyde dehydrogenase 1 (ALDH1), a soluble protein is used to detect CSC in various cancers, including leukemia [18], breast [19], colon [20], liver [21], lung [22] and pancreatic [23] cancers. In fact, the ALDH assay has served as a means to estimate stem cell features [24]. As CSC exhibit tumor growth and drug resistance, they provide a valuable model in which to investigate chemo-drug effects. Of particular relevance to the present report is the use of cisplatin in this CSC model. Cisplatin is a well-known anticancer drug used against a variety of malignancies, including laryngeal cancer [17].

Serum interleukin-6 (IL-6) levels are increased in laryngeal cancer patients as compared with healthy volunteers, and these serum levels show further increases as a function of malignancy progression [25, 26]. Elevated levels of IL-6 are also observed in tissue specimens of laryngeal cancer [25]. Secretion of IL-6 has been suggested to act as a potential biomarker for assessing the aggressive tumor phenotype in laryngeal carcinoma. Findings from recent studies have indicated that the expressions of CSC markers are significantly upregulated in IL-6 expressing lung cancer cells and cell-derived tumor xenograft tissues after cisplatin treatment. However, these CSC markers were not upregulated in IL-6 knockdown cells and in IL-6 knockdown cell-derived tumor tissue [27]. Negative effects of IL-6 signaling in triggering increased tumor growth and drug resistance in lung cancer during cisplatin treatment have been reported [17]. As signal transducer and activator of transcription 3 (STAT3) and hypoxia-inducible factor 1 (HIF1) are the downstream molecules of the IL-6 signaling pathway, STAT3 activation has been observed in cancers and its activation in tumor cells plays a crucial role in mediating and promoting tumorigenesis [28–32]. Moreover, hypoxia-inducible factor 1α (HIF-1α), as the core of the hypoxia-related response network [33], can bind to downstream molecules to induce the formation of angiogenesis and multidrug resistance genes [34]. While IL-6/STAT3/HIF1 signaling has been reported to play an important role in the treatment of ovarian cell cancer [35], the issue of whether the IL-6/STAT3/HIF1 pathway may play a role in laryngeal cancer remains uncertain.

In the present study, we aimed to explore whether IL-6 knockdown enhances the effectiveness of cisplatin in laryngeal CSCs and the potential involvement of IL-6/STAT3/HIF1 signaling. To accomplish this goal, we used hep2, the laryngeal squamous cancer cell line, and isolated ALDH+ and CD44+ CSC from hep2 cells along with siRNA technology to silence IL-6 gene expression. We observed that in response to IL-6-knockdown, laryngeal CSC characteristics show marked changes and enhanced effects of IL-6 knockdown on anti-tumor effects of cisplatin were demonstrated upon a number of parameters including cell proliferation, invasion, tumorigenesis, apoptosis and tumors in xenograft studies.

## Materials and methods

### Cell culture

A human laryngeal squamous cancer cell line, Hep2, was purchased from ATCC (Manassas, VA, USA), and cultured in Dulbecco’s modified Eagle’s medium /F12 supplemented with 10% fetal bovine serum. Cells were maintained at 37 °C in a humidified incubator with a mixture of 95% air (20% O_2_) and 5% CO_2_ environment. When applicable, cisplatin was used at an optimal dose of 5 μg/mL in cultured cells as suggested from our previous study [36].

### Fluorescence-activated cell sorting

Flow cytometry assays for the CD44+ and, subsequently ALDH+ cells were performed in this study. Briefly, Hep-2 cells were collected and rinsed with phosphate-buffered solution (PBS). The number of dissociated cells was counted, then treated with fluorochrome-conjugated CD44+ antibody for 30 min at 4 °C and protected from light. Once completed, cells were then washed and analyzed using a flow cytometer. The CD44+ and CD44− cells were sorted by the fluorescence-activated cell sorting (FACS) technique and the proportion of CD44+ cells were recorded. The CD44+ cells were further treated with PBS containing fluorochrome-conjugated ALDH+ antibody for 30 min at 4 °C. Once completed, cells were washed and sorted by FACS and analyzed for the proportion of ALDH+ and CD44+ cancer stem cells.

### IL-6 knockdown

IL-6 siRNA expression plasmids were purchased from Sigma-Aldrich (St Louis, MO). ALDH+/CD44+ Hep2-CSC were transfected with siRNA-IL-6 to knock down IL-6. The siRNA was transfected into the CSC using Lipofectamine 2000 (invitrogen life technologies) according to the manufacturer’s instructions. Total RNA was prepared 24 h post-transfection and the results of gene knockdown were determined by reverse transcription-quantitative polymerase chain reaction (RT-qPCR).

### MTT assay

Hep2-CSC cell proliferation with or without siRNA-IL-6 was measured with use of the MTT assay. In brief, MTT (20 μL) was added to each well of the plate and cells were incubated for 4 h at 37 °C. After incubation, DMSO (150 μL) was added to the well in the dark for 2 h to develop coloration. The absorbance values (490 nm) of each well were measured using an automatic multi-well spectrophotometer. Data were obtained from triplicate wells per condition and representatives of at least three independent experiments.

### Colony formation assay

Cell suspensions were diluted to a density of 200 cells per culture plate and then placed in the incubator for 2 weeks. Incubation was terminated when the colonies were visually perceptible. The colonies were then fixed in 1:3 acetic acid/methanol for 15 min and stained with Giemsa staining solution for 10–30 min. The number of colonies was counted when viewed microscopically.

### Cell invasion assay

The effect of cisplatin with/without siRNA-IL-6 on the invasion of Hep2-CSC was analyzed using Boyden chambers with coated Matrigel as instructed by the manufacturer (BD Biosciences, San Jose, CA). The invasive cancer cells were stained with crystal violet and visualized microscopically. All experiments were performed at least twice in triplicates.

### Apoptosis

Apoptotic cells were measured with use of Annexin V/PI double staining. Briefly, cells were harvested in 0.25% trypsin, washed with PBS, resuspended in 250 µL of binding buffer and adjusted to 1 × 10^6^/mL. Staining solution containing annexin V/FITC and propidium iodide was added to the cell suspension. After incubation for 30 min at room temperature in the dark, cells were analyzed by flow cytometry (FACSAria, Becton–Dickinson, USA).

### Quantitative real-time PCR

Total RNA was extracted from cells or tumor tissue using the RNeasy Mini kit (Qiagen) according to the manufacturer’s instructions. Quantitative real-time PCR experiments were performed using appropriate primers and SYBR green power master mix (applied biosystems) to determine the mRNA expression levels of genes of interest. GAPDH served as a reference gene to normalize other genes. The GAPDH, IL-6, STAT3 and HIF1 fragments were amplified using the following primer sequences, respectively:

GAPDH, forward 5′-GGTGGTCTCCTCTGACTTCAACA-3′ and reverse 5′-GTTGCTGTAGCCAAATTCGTTGT-3′;

IL-6, forward 5′-ACCTTCCAAAGATGGCTGAA-3′ and reverse 5′-GGCTTGTTCCTCACTACTCTCAA-3′;

STAT3, forward 5′-CTGGTGTCTCCACTGGTCTATCT-3′ and reverse 5′-AAACTTGGTCTTCAGGTATGGG-3′

HIF1, forward 5′-CATCTCCATCTCCTACCCACA-3′; reverse 5′-CTGCTCTGTTTGGT GAGGC-3′.

### Western blot

Protein levels of interested targets were measured using Western blot. Briefly, cells or tumor tissues were homogenized and diluted with RIPA lysis buffer (50 mM Tris–Cl at pH 7.5, 150 mM NaCl, 1% NP-40, 0.5% sodium deoxycholate, 1 mM EDTA, 1 μg/mL leupeptin, 1 μg/mL aprotinin, 0.2 mM PMSF). Lysates containing equal amounts of proteins (20–40 μg) were separated on 8–10% SDS/PAGE gel and then transferred onto PVDF membranes (Millipore, Billerica, MA, USA). After blocking, membranes were incubated with primary antibodies for IL-6, STAT3, HIF1 or β-Actin and horseradish peroxidase-conjugated secondary antibodies (1:5000). Immunoreactive proteins were visualized in Imager (Bio-Rad) using the ECL system (Thermo Fisher Scientific, Rochester, NY, USA).

### In vivo xenograft studies

Tumor growth of xenografts was examined as developed from the ALDH+/CD44+ CSC with/without siRNA-IL-6. The cells (5 × 10^5^ cells in 100 µL of PBS) were subcutaneously injected into the right dorsal area of nude mice. When tumor size achieved approximately 150 mm^3^, animals were randomly assigned into one of the following four groups (N = 5/group): (1) CSC, (2) CSC + IL-6-siRNA, (3) CSC + cisplatin and (4) CSC + IL-6-siRNA + cisplatin. Cisplatin was administered to the mice via an intraperitoneal injection at 10 mg/kg daily for 10 days. Tumor development was monitored daily. Drug toxicity effects, such as weight loss, behavioral change and feeding pattern were continuously monitored during the treatment period. At the end of the experiment, mice were euthanized and tumor tissues were removed for determination of gene or protein expression levels in the tissue. All animal studies were performed in accordance with the recommendations in the guide for the care and use of laboratory animals of the national institute of health. The protocol was approved by the Institutional Animal Care and Use Committee of Binzhou Medical University.

### Immunohistochemistry

After dissection, tumor tissues were fixed in 2% paraformaldehyde overnight at 4 °C and then soaked in 30% sucrose solution for an additional 4 h at 4 °C. The frozen tumors were cut at 8 μm thickness by MICROM cryostat (MICROM International, Walldorf, Germany) and examined for the levels of targeted proteins. Briefly, sections were washed in PBS and incubated in the blocking buffer followed by primary antibodies for IL-6, STAT3 or HIF1. Sections were then incubated with the secondary antibody. The 3,3'-Diaminobenzidine was used as a substrate for staining. Staining was observed, photographed and density measured.

### Statistical analysis

The data were presented as the mean ± SEM. Differences in mean values between two groups were analyzed by two-tailed Student’s t-tests. Differences in three or more than three groups were analyzed by one-way ANOVA, followed by the post hoc Fisher’s least significant difference test. A p < 0.05 was required for results to be considered statistically significant.

## Results

### IL-6, STAT3 and HIF1 mRNA and protein expressions in Hep2-CSC

**Fig. 1:**
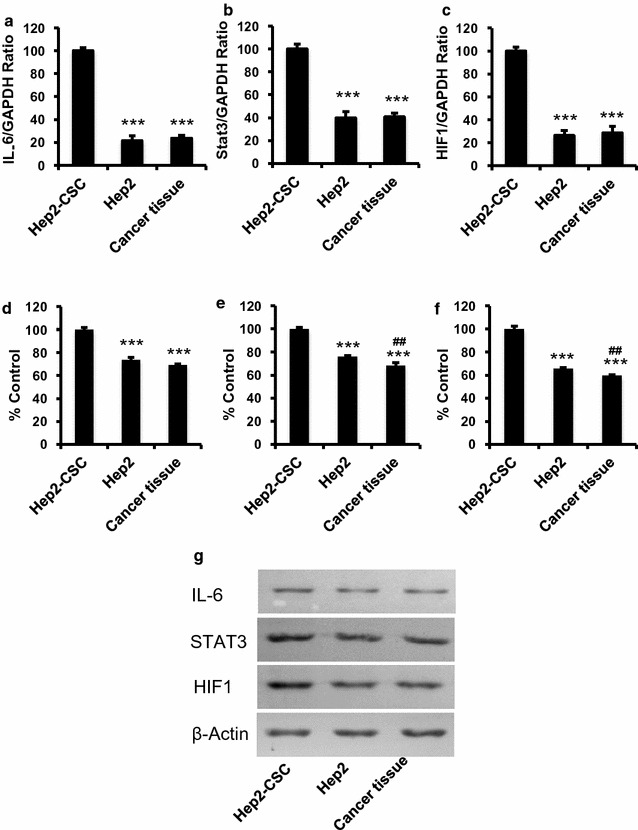
Increased IL-6, STAT3 and HIF1 mRNA (**a**–**c**) and protein (**d**–**g**) levels in hep2-CSC as compared with those from the hep2 cells or laryngeal cancer tissues. The relative IL-6, STAT3 and HIF1 mRNA expression levels were significantly increased in hep2-CSC as compared to the hep2 cells or cancer tissues (p < 0.001). GAPDH served as a house-keeping gene. Consistently, IL-6, STAT3 and HIF1 protein levels were increased in hep2-CSC as compared to controls. Actin was used to normalize protein levels. **p < 0.01, ***p < 0.001 vs hep2-CSC control; ^##^p < 0.01 vs hep2

Similar to our previous study [36], a high yield of ALDH+/CD44+ was obtained in this current study. IL-6, STAT3 and HIF1 mRNA levels in Hep2-CSC were significantly increased as compared to the IL-6, STAT3 and HIF1 mRNA expression levels in Hep2 cells and Hep2-derived tumor tissue, which were obtained from the tumor tissue after Hep2-cells were injected into dorsal area of nude mice (p < 0.001, Fig. 1a–c). In addition, IL-6, STAT3 and HIF1 protein levels in Hep2-CSC were consistently and significantly increased as compared with that from Hep2 cells or Hep2-derived tumor tissue (p < 0.001, Fig. 1d–g). The protein levels were obtained from three independent experiments and were compared among the groups after being normalized to β-actin levels.

### IL-6 siRNA effects upon mRNA and protein levels of IL-6, STAT3 and HIF1

**Fig. 2:**
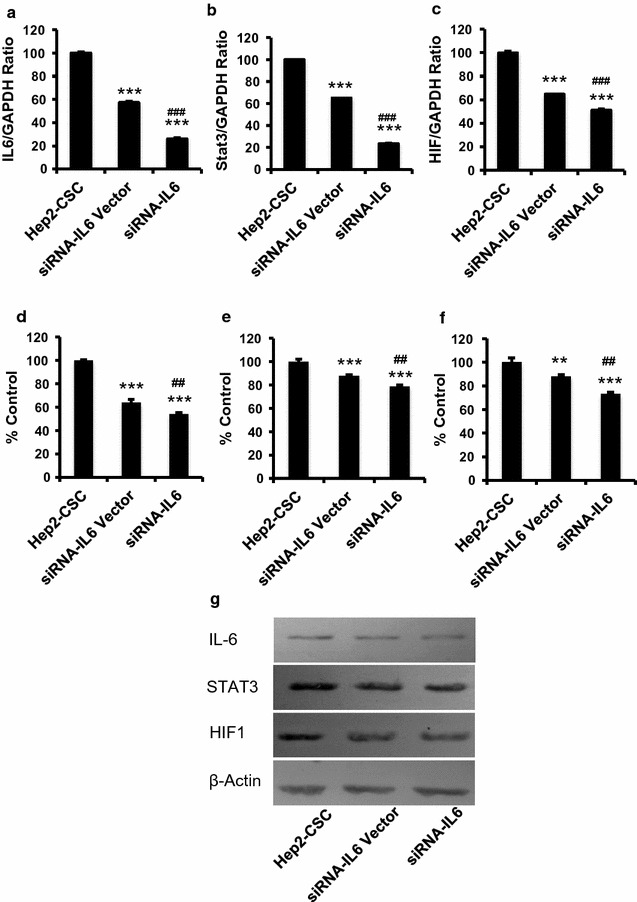
The siRNA-IL-6 knockdown reduced IL-6, STAT3 and HIF1 mRNA (**a**–**c**) and protein (**d**–**g**) levels in hep2-CSC. As compared with that of the corresponding mRNA of controls in hep2-CSC, IL-6, STAT3 and HIF1 mRNA levels were significantly decreased in the siRNA-IL-6 group (p < 0.05). The empty vector control group also showed diminished levels of IL-6, STAT3 and HIF1 mRNA. GAPDH served as a house-keeping gene. Similarly, IL-6, STAT3 and HIF1 protein levels were reduced significantly after siRNA-IL-6 treatment. Actin was used to normalize protein levels. ***p < 0.001 vs hep2-CSC control: ^###^p < 0.001 vs siRNA-IL-6 vector control

As compared to mRNA levels of IL-6, STAT3 and HIF1 in unaltered Hep2-CSC, siRNA-IL-6 significantly reduced mRNA expression levels of IL-6, STAT3 and HIF1 (Fig. 2a–c, p < 0.001). Although the siRNA-IL-6 vector also affected gene expression levels as compared to Hep2-CSC, the levels of reduction were less than that of siRNA-IL-6 (p < 0.05, Fig. 2a–c). Consistently, IL-6, STAT3 and HIF1 protein levels were significantly decreased after siRNA-IL-6 administration in Hep2-CSC (p < 0.001). As compared with that of the siRNA-IL-6 empty vector control, IL-6, STAT3 and HIF1 protein levels were significantly lower than that of the siRNA-IL-6 group (p < 0.01, Fig. 2d–g).

### siRNA-IL-6 knockdown enhances inhibitory effects of cisplatin on colony-formation and cell invasion

**Fig. 3:**
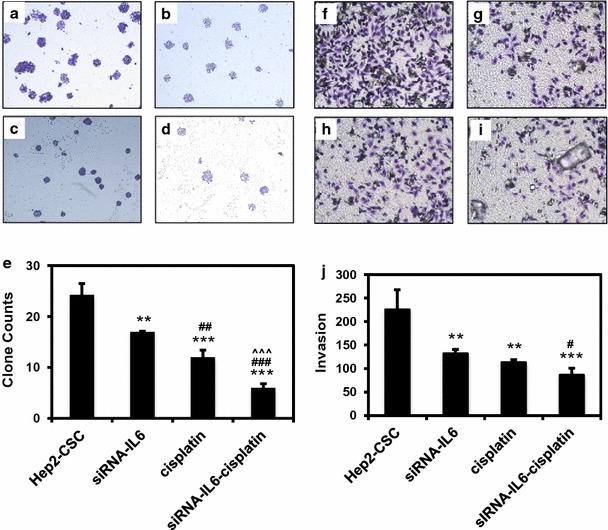
Colony formation (**a**–**e**) and invasion (**f**–**j**) results after siRNA-IL-6 knock down. As compared to the hep2-CSC group, siRNA-IL-6 or cisplatin treated hep2-CSC contained fewer cell colonies. The lowest degree of colony formation was observed when cisplatin treatment was combined with siRNA-IL-6. The siRNA-IL-6 or cisplatin treated hep2-CSC showed less invasion as compared with the hep2-CSC control. Again, the combination of cisplatin treatment with siRNA-IL-6 produced the lowest amount of invasion among the four groups. **p < 0.01, ***p < 0.001 vs hep2-CSC control; ^#^p < 0.05, ^##^p < 0.01, ^###^p < 0.001 vs siRNA-IL6 group; ^^^p < 0.001 vs cisplatin group

The in vitro tumorigenicity of hep2-CSC with/without IL-6-siRNA plus cisplatin was determined using a soft-agar assay. Fewer colonies were formed in the hep2-CSC cells treated with siRNA-IL-6 or cisplatin as compared to their corresponding vector controls. Maximal colony reduction was obtained when IL-6 siRNA was combined with cisplatin as compared to that observed with cisplatin or siRNA-IL-6 alone (p < 0.001, Fig. 3a–e). In addition, while siRNA-IL-6 knockdown or cisplatin both inhibited cell invasion when used alone, significantly greater reductions in cell invasion were obtained when cisplatin was combined with siRNA-IL-6 as compared with effects resulting from their individual application (p < 0.001, Fig. 3f–j).

### siRNA-IL-6 knockdown enhances inhibitory effects of cisplatin on cell proliferation

**Fig. 4:**
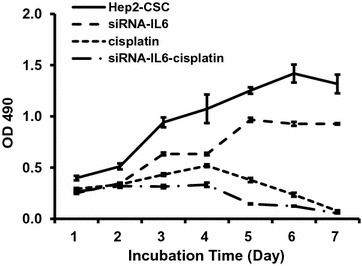
Proliferation results after siRNA-IL-6 knock down. Cisplatin or siRNA-IL-6 significantly inhibited Hep2-CSC cell proliferation in a temporally-dependent manner. The combined treatment of cisplatin and siRNA-IL-6 significantly enhanced the inhibitory effect of siRNA-IL-6. *p < 0.05, **p < 0.01 vs hep2-CSC control

To test the effect of siRNA-IL-6 on cell proliferation, Hep2-CSC cells exhibiting stable expressing control vectors, were exposed to either siRNA-IL-6, 10 µM cisplatin or siRNA-IL-6 combined with 10 µM cisplatin and examined with use of a MTT assay. Cisplatin or siRNA-IL-6 significantly inhibited Hep2-CSC cell proliferation in a temporally-dependent manner. However, their combined treatment substantially enhanced this inhibitory effect and produced the lowest cell proliferation rates as compared with that of the controls (Fig. 4).

### IL-6 knockdown enhances cisplatin mediated apoptotic effects

**Fig. 5:**
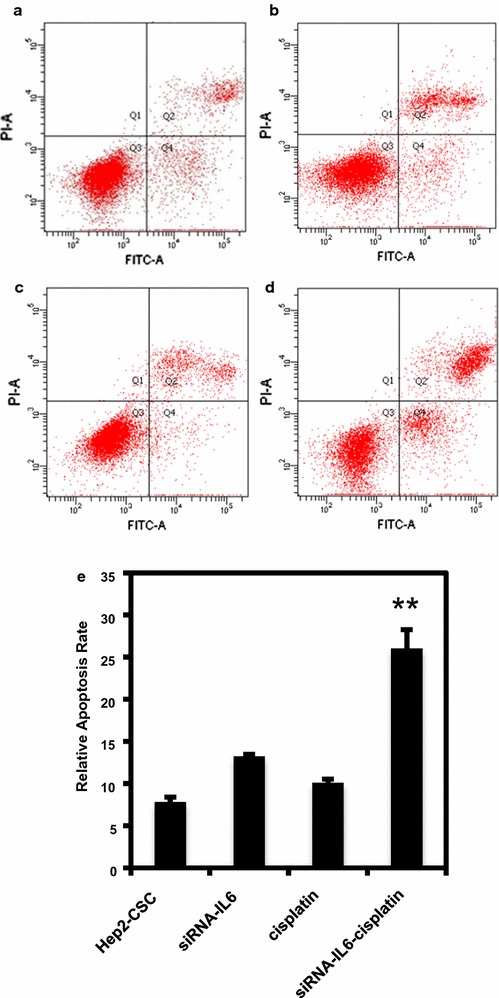
Cisplatin increases the pro-apoptotic effect of siRNA-IL-6. Representative images showing apoptosis in control (**a**), siRNA-IL-6-treated (**b**), cisplatin-treated (**c**), and siRNA-IL-6 and cisplatin-treated (**d**) hep2-CSC. Relative apoptosis rates (**e**) were slightly promoted by siRNA-IL-6 or cisplatin-treated hep2-CSC. The combination of cisplatin and siRNA-IL-6 significantly increased the pro-apoptotic effect of siRNAIL-6 or cisplatin. **p < 0.01 vs hep2-CSC control

To evaluate siRNA-mediated apoptotic effects, Hep2-CSC cells exhibiting stable expressing control vectors, were exposed to either siRNA-IL-6, cisplatin or siRNA-IL-6 combined with cisplatin and were subjected to FACS analysis. The siRNA-IL-6 or cisplatin alone resulted in similar rates of apoptosis as that seen in controls, while siRNA-IL-6 combined with cisplatin significantly enhanced cell apoptosis rates compared with controls (p < 0.01, Fig. 5).

### Cisplatin combined with IL-6 knockdown enhances antitumor effects in xenografts

**Fig. 6:**
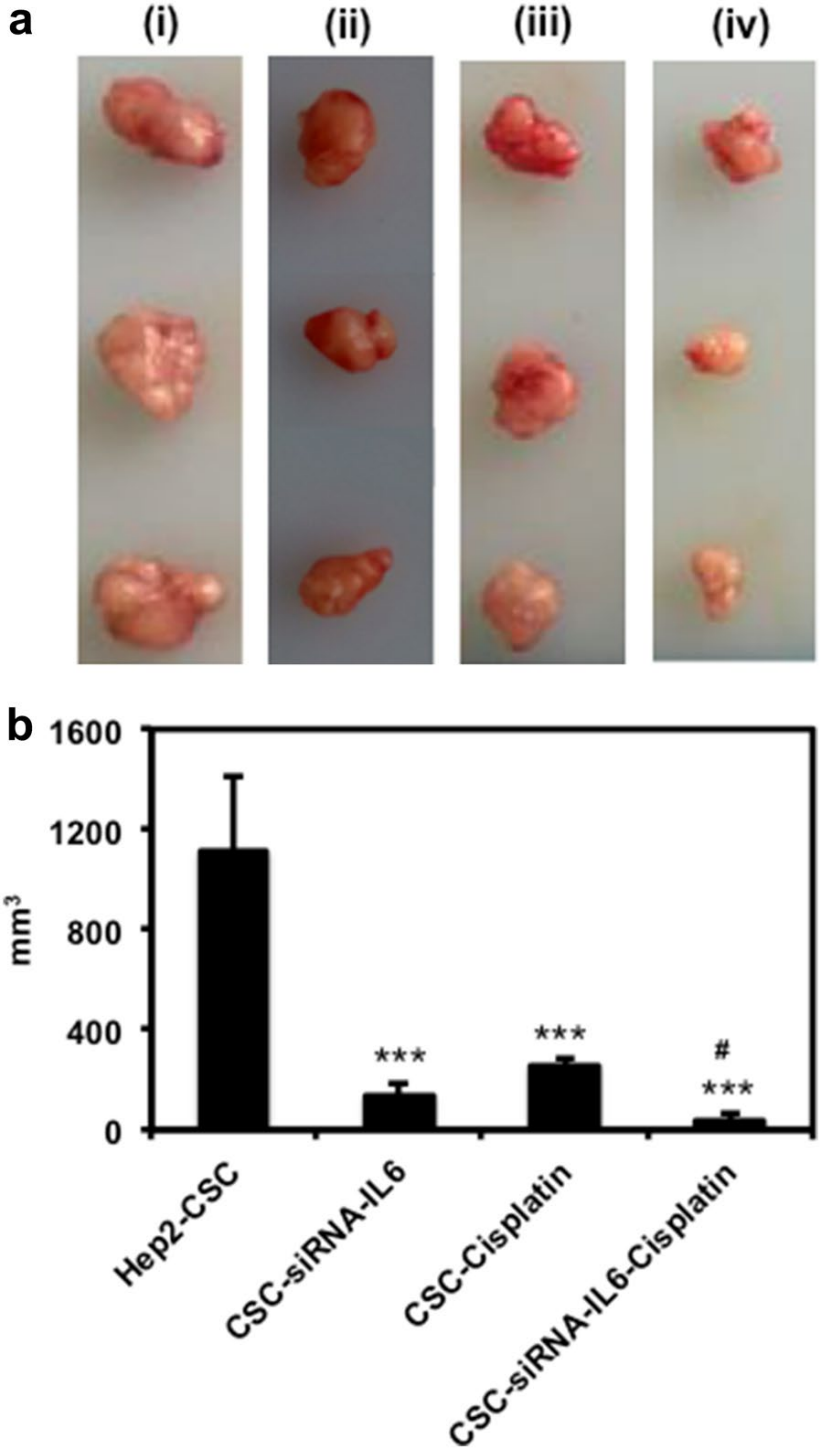
Xenograft cancer tissues (**a**) derived from hep2-CSC (i), hep2-CSC treated with siRNA-IL-6 (ii), hep2-CSC treated with cisplatin (iii) and hep2-CSC treated with siRNA-IL-6 and cisplatin (iv). Measure of tissue size (**b**) indicated that tumor growth was significantly decreased by siRNA-IL-6, cisplatin or the combined treatments, as compared to the hep2-CSC control. ***p < 0.001 vs CSC control; ^#^p < 0.05 vs CSC-Cisplatin group

We next examined tumor growth of xenografts developed from CSC cells with/without siRNA-IL-6 knockdown. Our data show that siRNA-IL-6 or cisplatin injection slowed tumor development, with tumor sizes in these groups being much smaller than that of the CSC group. Maximal reductions in tumor size were observed in the siRNA-IL-6 knockdown + cisplatin treated group (p < 0.001, Fig. 6).

### Decreased IL-6, STAT3 and HIF1 protein levels after siRNA-IL-6 knockdown in xenografts

**Fig. 7:**
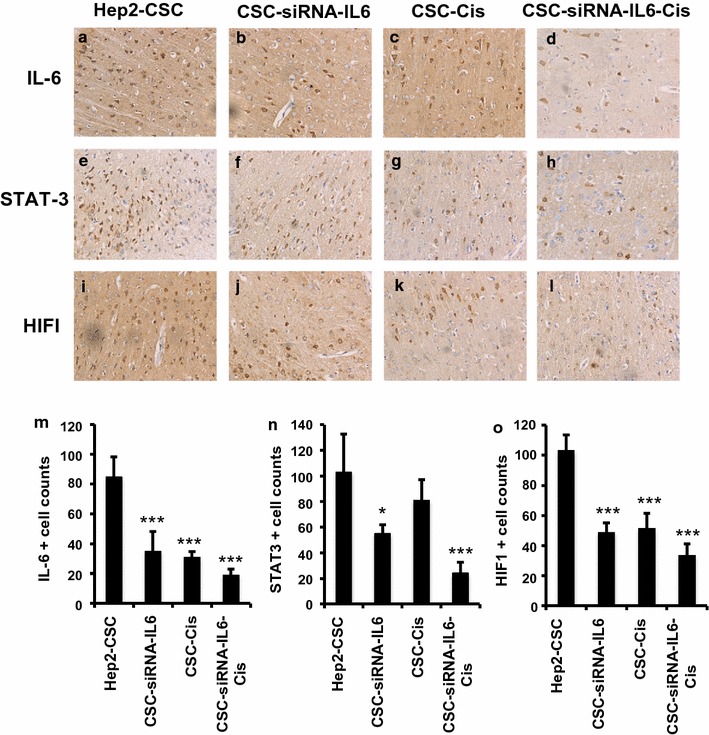
IL-6 (**a**–**d**), STAT3 (**e**–**h**) and HIF1 (**i**–**l**) protein levels as detected with immunohistochemistry in xenograft cancer tissues derived from hep2-CSC, hep2-CSC-siRNA-IL-6, hep2-CSC + cisplatin and hep2-CSC-siRNA-IL-6 + cisplatin. Image analysis results showed that the number of IL-6+, STAT3+ and HIF1+ cells (**m**–**o**) was decreased in response to siRNA-IL-6 or cisplatin-treated xenografts. Maximal reductions in IL-6+, STAT3+ and HIF1+ cell numbers were obtained in the group receiving the combined treatment of siRNA-IL-6 and cisplatin. *p < 0.05, ***p < 0.001 vs hep2-CSC control

Immunohistochemistry staining demonstrated lower numbers of positive-stained IL-6, STAT3 and HIF1 cells in tumor tissues developed from siRNA-IL-6 knockdown cells, as compared with that of the CSC-derived xenografts. The IL-6+, STAT3+ and HIF1+ cell numbers were also decreased in the cisplatin-treated xenografts. The siRNA-IL-6 + cisplatin group showed the fewest number of IL-6+, STAT3+ and HIF1+ cells (p < 0.001, Fig. 7).

## Discussion

Using in vitro cell lines and an in vivo xenograft model, we investigated whether knockdown of IL-6, as achieved using a siRNA technique, can increase the chemo-drug efficacy of cisplatin in laryngeal cancer. Our results show that siRNA-IL-6 combined with cisplatin reduced cell proliferation, colony formation and invasion and increased apoptosis to a greater degree than that obtained when either siRNA-IL-6 or cisplatin were administered alone. Similarly, results from our xenograft study showed greater efficacy upon suppressing the rate of tumor growth when siRNA-IL-6 was combined with cisplatin as compared with siRNA-IL-6 or cisplatin treatment alone. Taken together, our results suggest that IL-6 knockdown can increase chemo-drug efficacy, reduce drug resistance, inhibit tumor growth and reduce the potential for tumor recurrence and metastasis in laryngeal cancer. These siRNA-IL-6 effects were accompanied with decreased STAT3 and HIF1 mRNA and protein levels.

IL-6 levels in serum and cancer tissue are increased in laryngeal cancer patients as compared with healthy volunteers [25, 26], suggesting that IL-6 can act as a potential biomarker for assessing tumor growth and malignancy progression. However, IL-6 has also been found to alter the susceptibility of tumor cells to apoptosis by chemotherapeutic drugs [37]. Results from a recent study have revealed that IL-6 treatment was found to be associated with increased cisplatin resistance in lung CSC and increased CSC stemness [27]. When lung CSC were treated with neutralizing IL-6 antibody, cisplatin resistance decreased [27]. Using CSC from a different cancer source, we found that silencing IL-6 gene expression with siRNA significantly enhanced the cisplatin effect in laryngeal tumor cells as indicated by reductions in cell proliferation, colony formation, invasion, and an increase in the number of apoptotic cells. Accordingly, with the use of CSC, which is considered a very effective model for investigating drug-resistance, we demonstrate the importance of IL-6 signaling in triggering increased cisplatin efficacy in laryngeal cancer. In specific, we show that reducing IL-6 is beneficial for cisplatin efficacy particularly in a drug-resistant condition.

In this study, we identified and isolated ALDH+ and CD44+ CSC from laryngeal cancer cells as an approach to better predict the role of IL-6 in chemo-drug resistance. Cancer stem cell theory is one of the most likely explanations for chemoresistance and recurrence in cancer [38, 39]. Resistance of CSC to conventional therapies has been shown to result from multiple mechanisms [40], including increased expression of detoxifying enzymes such as ALDH. With the administration of chemotherapy and irradiation, ALDH alters aldehydes (oxygen, carbon, and hydrogen) within a cell to prevent DNA damage. Increased ALDH enzyme activity has been found in CSC derived from colon, ovarian, prostate, and breast cancers [41, 42]. ALDH+ CSC has also been found to mediate metastasis and result in poor clinical outcomes in inflammatory breast cancer [41], as well as predict engraftment of primary breast tumors [42].

This effect of IL-6 on cisplatin efficacy was accompanied by decreased STAT3 in these laryngeal cancer cells. The IL-6 signaling factor induces STATs tyrosine-phosphorylation and initiation by activating members of the janus kinase (JAK) family [43–45]. STAT3 is required and essential for tumorigenesis as shown in a variety of cancers. STAT3 has been reported to play a pivotal role in maintenance of stem cell-like breast cancer cells, which have been shown to be related to tumor recurrence, metastasis and chemo-resistance [28, 46]. In addition, STAT3 has been shown to be constitutively activated or over expressed in head and neck squamous cell carcinoma [47] and lung [48] cancers. Our results show that IL-6 and STAT3 expressions are increased in laryngeal CSC and decreased after IL-6 knockdown. These findings are consistent with what has been observed in prostate cancer, and blocking of STAT3 suppresses clonogenicity in stem cell-like cells from high grade prostate cancer patients [49]. STAT3 has also been reported to be involved in IL-6-induced proliferation of renal cancer cells [50].

The findings that HIF1 is increased in laryngeal CSC may indicate another factor that contributes to drug-resistance in CSC. HIF1 is comprised of α subunit which is oxygen-dependent and β subunit which is continually expressed. Under normal oxygen pressure/conditions, α subunit is rapidly degraded by the proteasome pathway, while under hypoxic conditions this subunit remains stable. Activated HIF-1α, when transferred into the nucleus, binds to downstream molecules, such as the anti-apoptotic factors Bcl-2, Survivin and Xiap. These anti-apoptotic factors can then induce the formation of angiogenesis and multi-drug resistance genes [34]. We found that the enhanced cisplatin efficacy after knockdown of IL-6 with siRNA was accompanied with decreased HIF1 levels. These results suggest that this reduction in HIF1 may be important component for increased cisplatin efficacy in laryngeal cancer.

## Conclusions

To the best of our knowledge, our study represents the first to examine the effect of IL-6 knockdown in combination with cisplatin in drug-resistance laryngeal cancer using ALDH+ and CD44+ CSC. Due to the limitations of cancer chemotherapy resulting from drug resistance, siRNA-based therapeutics has emerged as a promising new anticancer tactic. A small number of Phase I clinical trials that have been completed [51] and discussions regarding the benefits and limitations of siRNA for cancer therapy have been ongoing [39, 52, 53]. The results of our present study demonstrate distinct beneficial effects of IL-6 knockdown in combination with cisplatin treatment, and provide a theoretical base for applying siRNA techniques in the treatment of laryngeal cancer.

## Abbreviations

CSC: cancer stem cells
LSCC: laryngeal squamous carcinoma
ALDH: aldehyde dehydrogenase
HIF1: hypoxia-inducible factor 1
HNSCC: head and neck squamous cell carcinoma
IL-6: interleukin-6
STAT3: signal transducer and activator of transcription 3
HIF1: hypoxia-inducible factor 1
JAK: janus kinase
FACS: fluorescence-activated cell sorting
PBS: phosphate buffer solution

## References

[1] Jemal A, Bray F, Center MM, Ferlay J, Ward E, Forman D (2011). Global cancer statistics. CA Cancer J Clin.

[2] Ragin CC, Modugno F, Gollin SM (2007). The epidemiology and risk factors of head and neck cancer: a focus on human papillomavirus. J Dent Res.

[3] Jemal A, Siegel R, Ward E, Murray T, Xu J, Thun MJ (2007). Cancer statistics, 2007. CA Cancer J Clin.

[4] Marioni G, Marchese-Ragona R, Cartei G, Marchese F, Staffieri A (2006). Current opinion in diagnosis and treatment of laryngeal carcinoma. Cancer Treat Rev.

[5] Al-Hajj M, Clarke MF (2004). Self-renewal and solid tumor stem cells. Oncogene.

[6] Cho RW, Clarke MF (2008). Recent advances in cancer stem cells. Curr Opin Genet Dev.

[7] Lobo NA, Shimono Y, Qian D, Clarke MF (2007). The biology of cancer stem cells. Annu Rev Cell Dev Biol.

[8] Prince ME, Sivanandan R, Kaczorowski A, Wolf GT, Kaplan MJ, Dalerba P, Weissman IL, Clarke MF, Ailles LE (2007). Identification of a subpopulation of cells with cancer stem cell properties in head and neck squamous cell carcinoma. Proc Natl Acad Sci USA.

[9] Reya T, Morrison SJ, Clarke MF, Weissman IL (2001). Stem cells, cancer, and cancer stem cells. Nature.

[10] Wicha MS, Liu S, Dontu G (2006). Cancer stem cells: an old idea–a paradigm shift. Cancer Res.

[11] Tomao F, Papa A, Rossi L, Strudel M, Vici P, Lo Russo G, Tomao S (2013). Emerging role of cancer stem cells in the biology and treatment of ovarian cancer: basic knowledge and therapeutic possibilities for an innovative approach. J Exp Clin Cancer Res.

[12] Dean M, Fojo T, Bates S (2005). Tumour stem cells and drug resistance. Nat Rev Cancer.

[13] Donnenberg VS, Donnenberg AD (2005). Multiple drug resistance in cancer revisited: the cancer stem cell hypothesis. J Clin Pharmacol.

[14] Thapa R, Wilson GD (2016). The importance of CD44 as a stem cell biomarker and therapeutic target in cancer. Stem Cells Int.

[15] Yan Y, Zuo X, Wei D (2015). Concise review: emerging role of CD44 in cancer stem cells: a promising biomarker and therapeutic target. Stem Cells Transl Med.

[16] Zhou JY, Chen M, Ma L, Wang X, Chen YG, Liu SL (2016). Role of CD44high/CD133high HCT-116 cells in the tumorigenesis of colon cancer. Oncotarget.

[17] Sim MW, Grogan PT, Subramanian C, Bradford CR, Carey TE, Forrest ML, Prince ME, Cohen MS (2016). Effects of peritumoral nanoconjugated cisplatin on laryngeal cancer stem cells. Laryngoscope.

[18] Cheung AM, Wan TS, Leung JC, Chan LY, Huang H, Kwong YL, Liang R, Leung AY (2007). Aldehyde dehydrogenase activity in leukemic blasts defines a subgroup of acute myeloid leukemia with adverse prognosis and superior NOD/SCID engrafting potential. Leukemia.

[19] Ginestier C, Hur MH, Charafe-Jauffret E, Monville F, Dutcher J, Brown M, Jacquemier J, Viens P, Kleer CG, Liu S (2007). ALDH1 is a marker of normal and malignant human mammary stem cells and a predictor of poor clinical outcome. Cell Stem Cell.

[20] Carpentino JE, Hynes MJ, Appelman HD, Zheng T, Steindler DA, Scott EW, Huang EH (2009). Aldehyde dehydrogenase-expressing colon stem cells contribute to tumorigenesis in the transition from colitis to cancer. Cancer Res.

[21] Ma S, Chan KW, Lee TK, Tang KH, Wo JY, Zheng BJ, Guan XY (2008). Aldehyde dehydrogenase discriminates the CD133 liver cancer stem cell populations. Mol Cancer Res.

[22] Jiang F, Qiu Q, Khanna A, Todd NW, Deepak J, Xing L, Wang H, Liu Z, Su Y, Stass SA (2009). Aldehyde dehydrogenase 1 is a tumor stem cell-associated marker in lung cancer. Mol Cancer Res.

[23] Rasheed ZA, Yang J, Wang Q, Kowalski J, Freed I, Murter C, Hong SM, Koorstra JB, Rajeshkumar NV, He X (2010). Prognostic significance of tumorigenic cells with mesenchymal features in pancreatic adenocarcinoma. J Natl Cancer Inst.

[24] Karatas OF, Suer I, Yuceturk B, Yilmaz M, Hajiyev Y, Creighton CJ, Ittmann M, Ozen M (2016). The role of miR-145 in stem cell characteristics of human laryngeal squamous cell carcinoma Hep-2 cells. Tumour Biol.

[25] Chen Z, Malhotra PS, Thomas GR, Ondrey FG, Duffey DC, Smith CW, Enamorado I, Yeh NT, Kroog GS, Rudy S (1999). Expression of proinflammatory and proangiogenic cytokines in patients with head and neck cancer. Clin Cancer Res.

[26] Hao W, Zhu Y, Zhou H (2013). Prognostic value of interleukin-6 and interleukin-8 in laryngeal squamous cell cancer. Med Oncol.

[27] Zhang F, Duan S, Tsai Y, Keng PC, Chen Y, Lee SO, Chen Y (2016). Cisplatin treatment increases stemness through upregulation of hypoxia-inducible factors by interleukin-6 in non-small cell lung cancer. Cancer Sci.

[28] Marotta LL, Almendro V, Marusyk A, Shipitsin M, Schemme J, Walker SR, Bloushtain-Qimron N, Kim JJ, Choudhury SA, Maruyama R (2011). The JAK2/STAT3 signaling pathway is required for growth of CD44(+)CD24(−) stem cell-like breast cancer cells in human tumors. J Clin Invest.

[29] Grivennikov S, Karin E, Terzic J, Mucida D, Yu GY, Vallabhapurapu S, Scheller J, Rose-John S, Cheroutre H, Eckmann L (2009). IL-6 and Stat3 are required for survival of intestinal epithelial cells and development of colitis-associated cancer. Cancer Cell.

[30] Gao SP, Mark KG, Leslie K, Pao W, Motoi N, Gerald WL, Travis WD, Bornmann W, Veach D, Clarkson B (2007). Mutations in the EGFR kinase domain mediate STAT3 activation via IL-6 production in human lung adenocarcinomas. J Clin Invest.

[31] Yu H, Kortylewski M, Pardoll D (2007). Crosstalk between cancer and immune cells: role of STAT3 in the tumour microenvironment. Nat Rev Immunol.

[32] Huang C, Yang G, Jiang T, Huang K, Cao J, Qiu Z (2010). Effects of IL-6 and AG490 on regulation of Stat3 signaling pathway and invasion of human pancreatic cancer cells in vitro. J Exp Clin Cancer Res.

[33] Liu Y, Song X, Wang X, Wei L, Liu X, Yuan S, Lv L (2010). Effect of chronic intermittent hypoxia on biological behavior and hypoxia-associated gene expression in lung cancer cells. J Cell Biochem.

[34] Middleton K, Jones J, Lwin Z, Coward JI (2014). Interleukin-6: an angiogenic target in solid tumours. Crit Rev Oncol Hematol.

[35] Anglesio MS, George J, Kulbe H, Friedlander M, Rischin D, Lemech C, Power J, Coward J, Cowin PA, House CM (2011). IL-6-STAT3-HIF signaling and therapeutic response to the angiogenesis inhibitor sunitinib in ovarian clear cell cancer. Clin Cancer Res.

[36] Liu T, Liu P, Li Y, Cui C, Dai L, Zhou X, Jin C, Fu Q (2017). Inhibition of STAT3 with shRNA enhances the chemosensitization of cisplatin in laryngeal carcinoma stem cells. Int J Exp Pathol.

[37] Yusuf RZ, Duan Z, Lamendola DE, Penson RT, Seiden MV (2003). Paclitaxel resistance: molecular mechanisms and pharmacologic manipulation. Curr Cancer Drug Targets.

[38] Visvader JE, Lindeman GJ (2012). Cancer stem cells: current status and evolving complexities. Cell Stem Cell.

[39] Islam F, Gopalan V, Smith RA, Lam AK (2015). Translational potential of cancer stem cells: a review of the detection of cancer stem cells and their roles in cancer recurrence and cancer treatment. Exp Cell Res.

[40] Morrison R, Schleicher SM, Sun Y, Niermann KJ, Kim S, Spratt DE, Chung CH, Lu B (2011). Targeting the mechanisms of resistance to chemotherapy and radiotherapy with the cancer stem cell hypothesis. J Oncol.

[41] Charafe-Jauffret E, Ginestier C, Iovino F, Tarpin C, Diebel M, Esterni B, Houvenaeghel G, Extra JM, Bertucci F, Jacquemier J (2010). Aldehyde dehydrogenase 1-positive cancer stem cells mediate metastasis and poor clinical outcome in inflammatory breast cancer. Clin Cancer Res.

[42] Charafe-Jauffret E, Ginestier C, Bertucci F, Cabaud O, Wicinski J, Finetti P, Josselin E, Adelaide J, Nguyen TT, Monville F (2013). ALDH1-positive cancer stem cells predict engraftment of primary breast tumors and are governed by a common stem cell program. Cancer Res.

[43] Suzuki R, Sakamoto H, Yasukawa H, Masuhara M, Wakioka T, Sasaki A, Yuge K, Komiya S, Inoue A, Yoshimura A (1998). CIS3 and JAB have different regulatory roles in interleukin-6 mediated differentiation and STAT3 activation in M1 leukemia cells. Oncogene.

[44] Ihle JN, Witthuhn BA, Quelle FW, Yamamoto K, Thierfelder WE, Kreider B, Silvennoinen O (1994). Signaling by the cytokine receptor superfamily: JAKs and STATs. Trends Biochem Sci.

[45] Ihle JN, Kerr IM (1995). Jaks and Stats in signaling by the cytokine receptor superfamily. Trends Genet.

[46] Wang X, Wang G, Zhao Y, Liu X, Ding Q, Shi J, Ding Y, Wang S (2012). STAT3 mediates resistance of CD44(+)CD24(−/low) breast cancer stem cells to tamoxifen in vitro. J Biomed Res.

[47] Liu S, Ye D, Wang T (2017). Repression of GPRC5A is associated with activated STAT3, which contributes to tumor progression of head and neck squamous cell carcinoma. Cancer Cell Int.

[48] Alexandrow MG, Song LJ, Altiok S, Gray J, Haura EB, Kumar NB (2012). Curcumin: a novel Stat3 pathway inhibitor for chemoprevention of lung cancer. Eur J Cancer Prev.

[49] Kroon P, Berry PA, Stower MJ, Rodrigues G, Mann VM, Simms M, Bhasin D, Chettiar S, Li C, Li PK (2013). JAK-STAT blockade inhibits tumor initiation and clonogenic recovery of prostate cancer stem-like cells. Cancer Res.

[50] Horiguchi A, Oya M, Marumo K, Murai M (2002). STAT3, but not ERKs, mediates the IL-6-induced proliferation of renal cancer cells, ACHN and 769P. Kidney Int.

[51] Zuckerman JE, Davis ME (2015). Clinical experiences with systemically administered siRNA-based therapeutics in cancer. Nat Rev Drug Discov.

[52] Wu D, Han H, Xing Z, Zhang J, Li L, Shi W, Li Q (2016). Ideal and reality: barricade in the delivery of small interfering RNA for cancer therapy. Curr Pharm Biotechnol.

[53] Young SW, Stenzel M, Yang JL (2016). Nanoparticle-siRNA: a potential cancer therapy?. Crit Rev Oncol Hematol.

